# Magnetite nanoparticles enhance the performance of a combined bioelectrode-UASB reactor for reductive transformation of 2,4-dichloronitrobenzene

**DOI:** 10.1038/s41598-017-10572-y

**Published:** 2017-09-04

**Authors:** Caiqin Wang, Lu Ye, Jie Jin, Hui Chen, Xiangyang Xu, Liang Zhu

**Affiliations:** 1 Department of Environmental Engineering, Zhejiang University, Hangzhou, 310058 China; 2 Zhejiang Province Key Laboratory for Water Pollution Control and Environmental Safety, Hangzhou, 310058 China

## Abstract

Direct interspecies electron transfer (DIET) among the cometabolism microbes plays a key role in the anaerobic degradation of persistent organic pollutants and stability of anaerobic bioreactor. In this study, the COD removal efficiency increased to 99.0% during the start-up stage in the combined bioelectrode-UASB system (R1) with magnetite nanoparticles addition, which was higher than those in the coupled bioelectrode-UASB (R2; 83.2%) and regular UASB (R3; 71.0%). During the stable stage, the increase of 2,4-dichloronitrobenzene (2,4-DClNB) concentration from 25 mg L^−1^ to 200 mg L^−1^ did not affect the COD removal efficiencies in R1 and R2, whereas the performance of R3 was deteriorated obviously. Further intermediates analysis indicated that magnetite nanoparticles enhanced the reductive dechlorination of 2,4-DClNB. High-throughput sequencing results showed that the functional microbes like *Syntrophobacter* and *Syntrophomonas* which have been reported to favor the DIET, were predominant on the cathode surface of R1 reactor. It is speculated that the addition of magnetite nanoparticles favors the cooperative metabolism of dechlorinating microbes and electricigens during 2,4-DClNB degradation process in the combined bioelectrode-UASB reactor. This study may provide a new strategy to improve the performance of microbial electrolysis cells and enhance the pollutant removal efficiency.

## Introduction

Microbial electrolysis cells (MECs) as an emerging electricity-mediated microbial bioelectrochemical technology, are developed originally for high-efficiency biological hydrogen production from wastewater^1^. In addition to hydrogen production^2–4^, MECs could also support several unfavorable biological/chemical reactions energetically, and the integration of MECs with anaerobic digestion is hoped to boost the removal of refractory pollutants such as nitrobenzene^5^, chloramphenicol^6^, polychlorobiphenyl^7^, fluoronitrobenzene^8^, azo dye^9–11^ and 2,4-dinitrochlorobenzene^12^.

Despite the advantages of anaerobic MECs system, several issues still need to be addressed. Volatile fatty acids (VFAs) accumulation during anaerobic digestion is a common challenge, because excess VFAs could result in anaerobic acidification and low mineralization rate of refractory organics such as chlorinated nitroaromatic compounds. Previous studies^13–15^ have shown that low electron transfer efficiency is the critical reason for low mineralization rate. At the same time, anaerobic MEC-system generates a vast and undesired electron sink, and the electrons from organic matters are transferred to various acceptors, which is essential to improve anaerobic digestion efficiency and the anodic oxidation rate^16^. It was reported that direct interspecies electron transfer (DIET) may be a more effective mechanism for interspecies electron exchange under anaerobic conditions than interspecies electron transfer via reduced molecules such as hydrogen and formate^15, 17^.

In recent years, the stimulated Fe(III) reduction has been demonstrated to enhance the degradation of VFAs and methane production significantly during anaerobic digestion^18^. Cost-effective electrode materials, which could realize the efficient electron transfer from microbes to electrode in MFCs, might be an alternative for MECs. Thereinto, the carbon fiber brush, carbon mesh and graphite felt are the major materials used as the electrode^1^. Kong *et al*.^19^ modified the electrodes configuration to be toric, and the performance of bioelectrochemical system was improved greatly, including pollutant removal and electrochemical characterization. When the cathodic electrode surrounded by anodic electrode, it may facilitate electron transfer and increase the active area of system, and the pollutant degradation efficiency was enhanced finally. However, the previous studies have rarely considered the cost of the MEC-anaerobic system for application, and the efficiency and stability of MEC-anaerobic system needs to be improved.

Several studies^20–24^ have demonstrated that conductive iron oxides, e.g., goethite, hematite and magnetite, could promote the anaerobic biological treatment of wastewater by enhancing the direct interspecies electron transfer between functional microbial species. According to the function of conductive iron oxides in the DIET, a novel combined bioelectrode-UASB reactor with magnetite addition has been developed in this study. Carbon fiber brush and graphite felt were chose as the anode and cathode respectively in the combined bioelectrode-UASB due to their cost-effectiveness and good stability, and the anodic electrode was surrounded by the cathode electrode. The objectives are to 1) study the degradation of refractory pollutants such as 2,4-dichloronitrobenzene (2,4-DClNB), stability of combined system, and to 2) analyze the biofilm characteristic on electrodes surface and microbial community structure in combined system for revealing the possible mechanism for enhancement of DIET by magnetite addition.

## Results and Discussion

### Startup performance of different reactors

#### COD removal efficiency and variation of pH

The reactors were inoculated with the sludge from a municipal wastewater treatment plant, which is running at low organic loading rate (0.4 kg m^−3^ d^−1^). With the increase of COD loading in the influent, the COD removal efficiencies of R2 and R3 were unstable during the first 45 days, whereas the COD removal efficiency in R1 increased from 41.8% to 99.0% (Fig. S1). Afterward, the COD removal efficiency in R2 increased to 83.2% while the corresponding value in R3 was 71.0%. The performance of R2 was not enhanced immediately which might be due to the slow formation rate of conductive biological networks electrically among anaerobic microorganisms^25^. Compared with the COD removal efficiencies of R2 and R3, the value of R1 was higher, indicating the enhancement of magnetite nanoparticles for COD removal. Results showed that the addition of magnetite particles may represent a novel strategy to alleviate the impact of shocks caused by sudden variations in the influent flow rate and substrate concentration, which often result in the accumulation of propionate and butyrate ultimately in incomplete methanogenesis^21^.

Accordingly, the effluent pH of R2 and R3 were at lower level (less than 6.0) during the first 45 days (Fig. S1(b)). After that, the effluent pH of R2 remained between 6.6 and 6.9 while the corresponding value of R3 was between 6.5 and 6.7. In contrast, the effluent pH of R1 was almost between 6.5 and 7.0 during the first 45 days, and then stayed between 6.8 and 7.2, which is optimum for methanogens growth. In short, the results indicated that magnetite nanoparticle supplementation in R1 contributed to the stability of pH. It is supposed that the enhancement of COD removal in R1 by magnetite nanoparticles addition resulted in lower concentration of intermediates, e.g. VFAs, which will lead to acidification. Theoretically, during the degradation of organics, a mass of carbon dioxide and bicarbonate will be produced. Besides, magnetite was proved to promote the methanogenesis in this study and the results were in accordance with other studies^20, 21^. Therefore, the methanogenesis process could increase alkalinity by consuming vast hydrion.

#### ORP and Current variation

**Figure 1:**
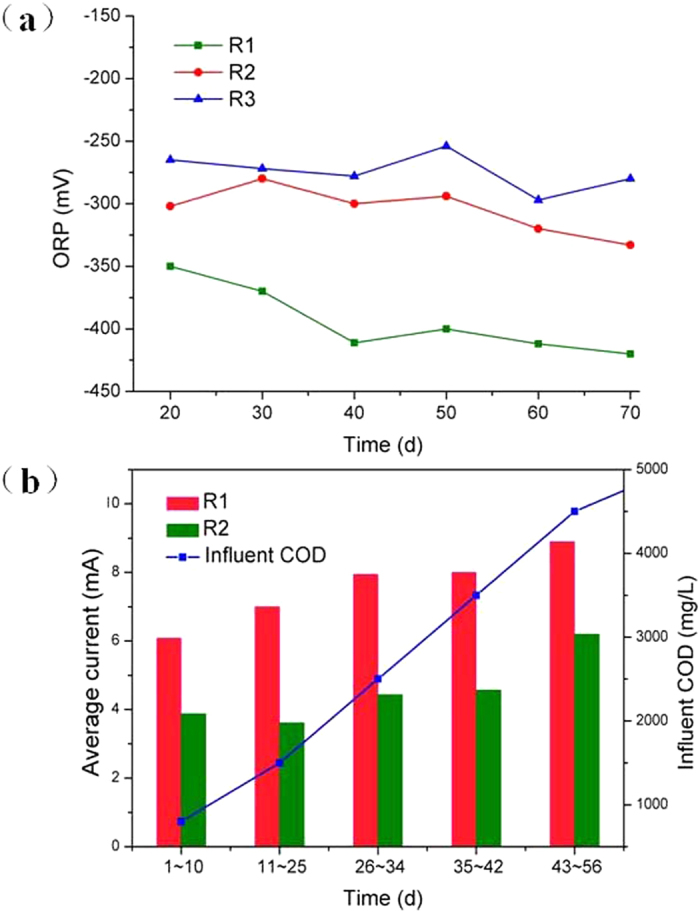
(**a**) ORP variation of different reactors during start-up stage (**b**) Current change in R1 and R2 during start-up stage.

As shown in Fig. 1(a), the ORP of R1 and R2 decreased from −340 mV to −420 mV and from −280 mV to −333 mV respectively, which were lower than that of R3 (from −250 mV to −300 mV). According to the previous researches^6, 8, 14^, the bioelectrochemical system can provide a better reductive condition with external power supply. With magnetite addition, the ORP was further reduced. This might be due to that the supplied magnetite nanoparticle worked as the electron acceptors^20, 26^. It was reported that insoluble Fe(III) oxides can accept the extra electrons from microbes, and they may serve as an electron chamber^22^. The effluent Fe^2+^/Fe^3+^ concentration of the three reactors were detected (Fig. S2). At the beginning, the Fe^2+^ concentration in the effluent of R1 was 1.75 times higher than that of R2, suggesting part of magnetite was reduced.

As the related researches, the lower ORP was suitable for methanogens growth^27^. Results showed that the combined bioelectrode-UASB system strengthened by magnetite nanoparticles provided a better growth environment for methanogens. At the same time, the contents of CH_4_ in the biogas produced in the three reactors were detected after start-up stage. The average CH_4_ contents in R1 (58.15 ± 1.01%) was 1.21 times and 1.35 times higher than those in R2 (48.15 ± 0.85%) and R3 (43.12 ± 0.91%), respectively.

Figure 1(b) indicated the current variation in R1 and R2 during start-up stage. When the influent COD loading increased from 0.8 kg m^−3^d^−1^ to 4.5 kg m^−3^d^−1^ gradually, the current increased in R1 and R2, indicating that more organics were oxidized on the anodes. Though supplied with the same voltage of 1.5 V, current in R1 was always higher than that in R2 which showed the electron transfer efficiency was enhanced by magnetite nanoparticles. The conductive magnetite might work as conduit^21, 23^ and promote the electrons transfer between microbes and electrodes^24^. Nakamura *et al*.^25^ reported that several microorganisms have the ability to self-assemble into an electrically conductive network in the presence of iron oxides, and magnetite nanoparticles might stimulate long-distance extracellular electron transfer in the microbial network.

#### VFAs levels in reactors

To better illustrate the effect of magnetite on the anaerobic acidification process, the effluent VFAs concentration was analyzed as depicted in Fig. S3. The results showed that the total VFAs concentrations of R1, R2 and R3 were 232.62 mg L^−1^, 670.28 mg L^−1^ and 919.4 mg L^−1^ respectively, indicating the acceleration of VFAs degradation by magnetite and extra electric field supplementation. Thermodynamically, propionate oxidation was inferior, and the reaction was as follows:

$${{\rm{CH}}}_{3}{{\rm{CH}}}_{2}{{\rm{COO}}}^{-}+3{{\rm{H}}}_{2}{\rm{O}}\to {{\rm{CH}}}_{3}{{\rm{COO}}}^{-}+{{{\rm{HCO}}}_{3}}^{-}+{{\rm{H}}}^{+}+3{{\rm{H}}}_{2}\,{\rm{\Delta }}G=76{\rm{kJ}}\,{{\rm{mol}}}^{-1}$$

This can only proceed if the hydrogen partial pressure is kept very low^28^. Among the three reactors, the effluent propionate concentration of R1 was the lowest at 73.19 mg L^−1^, followed by those of R3 (105.41 mg L^−1^) and R2 (224.34 mg L^−1^). Magnetite is assumed to act as a conduit for direct electron transfer from acidogens to methanogens^16, 20, 21^, which avoids the generation of intermediate hydrogen; thus, the hydrogen partial pressure is reduced, and the oxidation of propionate is strengthened^29^. Propionic-acid-type fermentation can happen when ORP was above −278 mV^4^. According to results in Fig. 1(a), propionate oxidation would be easier in R3 than in R2. This result is coincided with the phenomenon of propionic acid accumulation in R2.

#### Characteristics of anaerobic granular sludge

The mean granule sizes of R1, R2 and R3 reached 621.4 μm, 598.4 μm and 350.3 μm after 70-day operation, which were 5.76, 5.38 and 3.25 times higher than that of the seed sludge (107.9 μm), respectively. The results illustrated that electricity can stimulate microbial aggregation and magnetite has a tendency to further enhance the sludge granulation. It was supposed that with a large specific surface area and a positive charge, magnetite nanoparticles might play a role as a flocculant and could accelerate the sludge granulation by gathering the microbes with substrates. Moreover, some microorganisms have been demonstrated to access insoluble Fe (III) oxides by chemotaxis^23^.

Aulenta *et al*.^30^ found that part of magnetite would decompose into soluble Fe^2+^/Fe^3+^ in the anaerobic system. The result of effluent Fe^2+^/Fe^3+^ concentration (Fig. S2(a)) showed that, the total iron concentration in the effluent of R1 was 1.6 times higher than that of R2 at the beginning, suggesting the trace amount decomposition of magnetite. However, the total iron concentration in the effluent of R1 decreased gradually. It was supposed that the magnetite nanoparticles were incorporated in the sludge to remain in the reactor. After 70-day operation, the iron content of the sludge from each reactor was measured. The iron content in the sludge sample from R1 was the highest (74.33 mg Fe gVSS^−1^), and it was 19 times higher than those in R2 and R3 (Fig. S2(b)).

Microphotographs of the granular sludge from R1, R2 and R3 are shown in Fig. S4(b–d). Scanning electron microscope (SEM) was used to observe the structure of the granules, and the surface of granules was mainly comprised of filamentous bacteria (Fig. S4(e)), cocci-like (Fig. S4(f)) and bacilli-like (Fig. S4(g)). Based on the observation of sliced granules from R1, the magnetite nanoparticles were found to be wrapped tightly by microorganisms, suggesting that magnetite can be an essential factor to accelerate sludge granulation. Furthermore, SEM-EDX was used to observe the Fe distribution in the sludge from R1 (Fig. S5), and the iron was found to adhere to the surface of the microbes. The results support that magnetite can act as a conduit for electron transfer between microbes^21^.

### Reactor performance during stable stage

When the performance of R1, R2 and R3 remained stable, the experiment of stage two was carried out. The addition of 2,4-DClNB was increased gradually from 25 mg L^−1^ to 200 mg L^−1^ through four phases with the influent COD loading maintained at 5.0 kg COD m^−3^d^−1^.

#### Stability of different reactors

**Figure 2:**
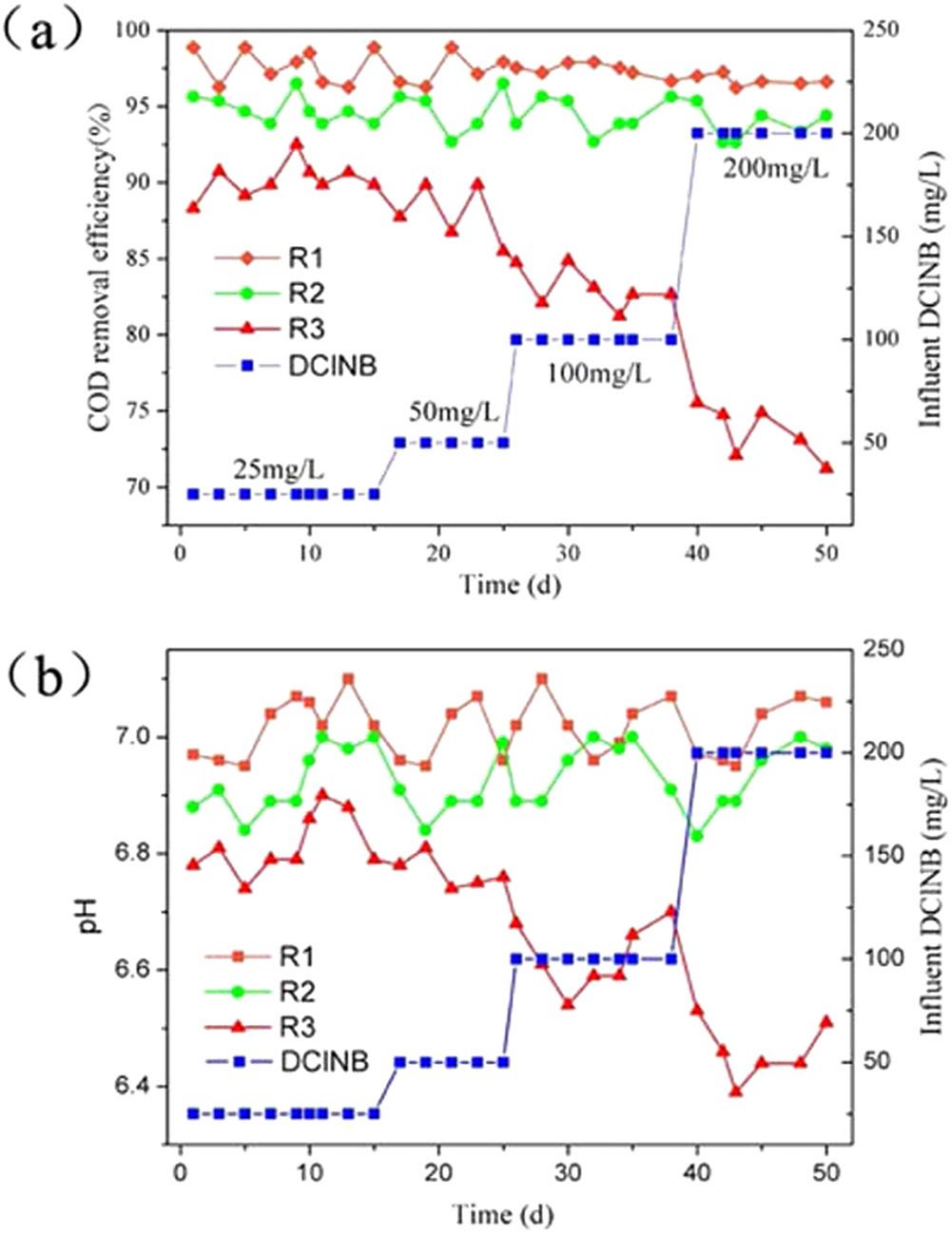
Effect of DClNB concentration on reactor performance: (**a**) COD removal efficiency (**b**) pH change.

The COD removal and pH variation of the three parallel reactors during stable stage are shown in Fig. 2. When the influent 2,4-DClNB was increased gradually from 25 mg L^−1^ to 200 mg L^−1^, the COD removal efficiency of R1 remained between 96.2% and 99.8%, whereas the corresponding value of R2 decreased from 94.6% to 84.3%. By contrast, the COD removal efficiency of R3 significantly decreased when the inflow 2,4-DClNB was increased to 100 mg L^−1^; when the 2,4-DClNB was further increased to 200 mg L^−1^, the effluent COD of R3 increased drastically from 584.5 mg L^−1^ to 1438.5 mg L^−1^ (the COD removal efficiency was 71.2%). Furthermore, the pH of the effluent from R1 and R2 was maintained at approximately 7.0, which was optimum for methanogens growth, whereas the one from R3 fluctuated and was even lower than 6.5.

The anaerobic microorganisms are very sensitive to the environmental condition. The addition of toxicant 2,4-DClNB might lead to the accumulation of acidity in the reactor, whereas the performance of R1 and R2 remained stable. A rational explanation is that with biological electrodes, R1 and R2 achieved a lower ORP in the anaerobic system which provided a better niche for the growth of anaerobes^31^.

#### Analysis of intermediate products

Despite the gradual increase of 2,4-DClNB loading from 25 g m ^−3^ d^−1^ to 200 g m^−3^ d^−1^, the pollutants were almost degraded totally in R1. When the pollutant loading reached 200 g m^−3^ d^−1^, the 2,4-DClNB removal efficiency in R3 decreased to 94.2 ± 1.9% (Fig. S6(a)). The formation of the intermediate products (including 2,4-DClAn, 4-ClAn and 2-ClAn) were analyzed using the mass balance method with the influent 2,4-DClNB maintained at 200 g m^−3^d^−1^. Compared with R3, no 2,4-DClNB was detected in the outflow of R1 and R2 (Fig. S6(b)), and the cumulative intermediates content of R1, R2 and R3 were 68.7%, 75.0% and 91.9% respectively, with 4-ClAn as the main intermediate. The outflow of R1 and R2 contained 2,4-DClAn and 4-ClAn, whereas the effluent of R3 contained one more intermediate, 2-ClAn; however, its content was much lower than that of 4-ClAn. This phenomenon showed that ortho chlorine was more likely to be removed than counterpoint chloride, which is consistent with the existing research^32^.

Considering the Cl^−^ formation (Table S1), the pollutants were further dechlorinated and mineralized in R1 and R2, leading to the lower concentration of intermediates. Compared with R3, the bioelectrode-combined anaerobic process showed a better dechlorination performance, and the magnetite could further strengthen the reductive dechlorination performance of the combined bioelectrode-UASB reactor.

The enhanced reduction of ClNBs in the bioelectrochemical system has been attributed to higher diversity of reduction-related species, potential electroactive species and fermentative species^12^. Aulenta *et al*.^24^ predicted that magnetite could promote the establishment of a syntrophic metabolism based on DIET between dechlorinating and other microorganisms. The coupled system was further enhanced by magnetite due to the construction of conductive biological networks^25, 33^.

#### Characteristic of electrodes

Graphs of the graphite fiber brush anodes taken by a scanning electron microscope (SEM) are shown in Fig. S7. Compared with the fresh graphite fiber brush (Fig. S7(a)), the anodic surfaces of the samples from R1 and R2 were covered with microbial aggregates which mainly consisted of bacilli-like and cocci-like. The anodic surface was covered by nano magnetite which might enhance the electron transport between the anode and the microbes. Except for direct adherence to the anode, many microbes clustered around the anode and connected to the graphite fiber brush via filamentous bacteria. A large amount of nano magnetite was observed in the microbe clusters. Thus, it is speculated that the magnetite prompted the formation of a conductive biological network around the anode, thereby facilitating the long-distance extracellular electron transfer between the free microorganisms and the electrode. By contrast, this phenomenon was not observed in the R2 reactor.

SEM graphs of cathodes in R1 and R2 (Fig. S8), both cathodic surfaces of R1 and R2 were covered by a layer of biofilm. This layer is supposed to accept the electrons directly from the cathode surface and participate in the process of methanogenesis or the reductive transformation process of the target pollutants. In addition, magnetite nanoparticles were found to attach to the surface of the microbe cells on the cathode of R1 tightly, which reflected that the magnetite nanoparticles might be involved in the electron transfer of functional microbes.

#### Analysis of microbial community

The microbial communities of seven samples from the three anaerobic reactors at the steady-stage condition and one sample from the seed sludge were analyzed by 454 high-throughput sequencing. The number of operational taxonomic units (OTUs) observed at a 3% distance is shown in Table S2, and the number of OTUs in R1 was higher than those in R2 and R3. The alpha diversity index is shown in Table S3, including Chao1, ACE estimator, and the Simpson and Shannon diversity indexes. As a metric for species richness, the total number of OTUs estimated by analyzing the Chao1 and ACE estimators were slightly higher in the reactor R1 (including Ano-R1, Cat-R1 and Sludge-R1) than those of R2 (including Ano-R2, Cat-R2 and Sludge-R2) and R3 (Sludge-R3). The Simpson and Shannon diversity indexes, which emphasize the species diversity and evenness of the community, increased notably both on the anode and on the cathode with the addition of magnetite nanoparticles, whereas Sludge-R1 and Sludge-R2 were similar (the Simpson index for both were 0.94, and Shannon index were 6.59 and 6.45, respectively). It is hypothesized that the intensity and potential of the electric field around the electrodes could be improved by adding nano magnetite, and then increases the microbial diversity in the combined system. A high microbial diversity could enhance the system stability, which could result in a high capacity to resist environmental stress^16^, such as influent pollutants loading shock (Fig. 2).

**Figure 3:**
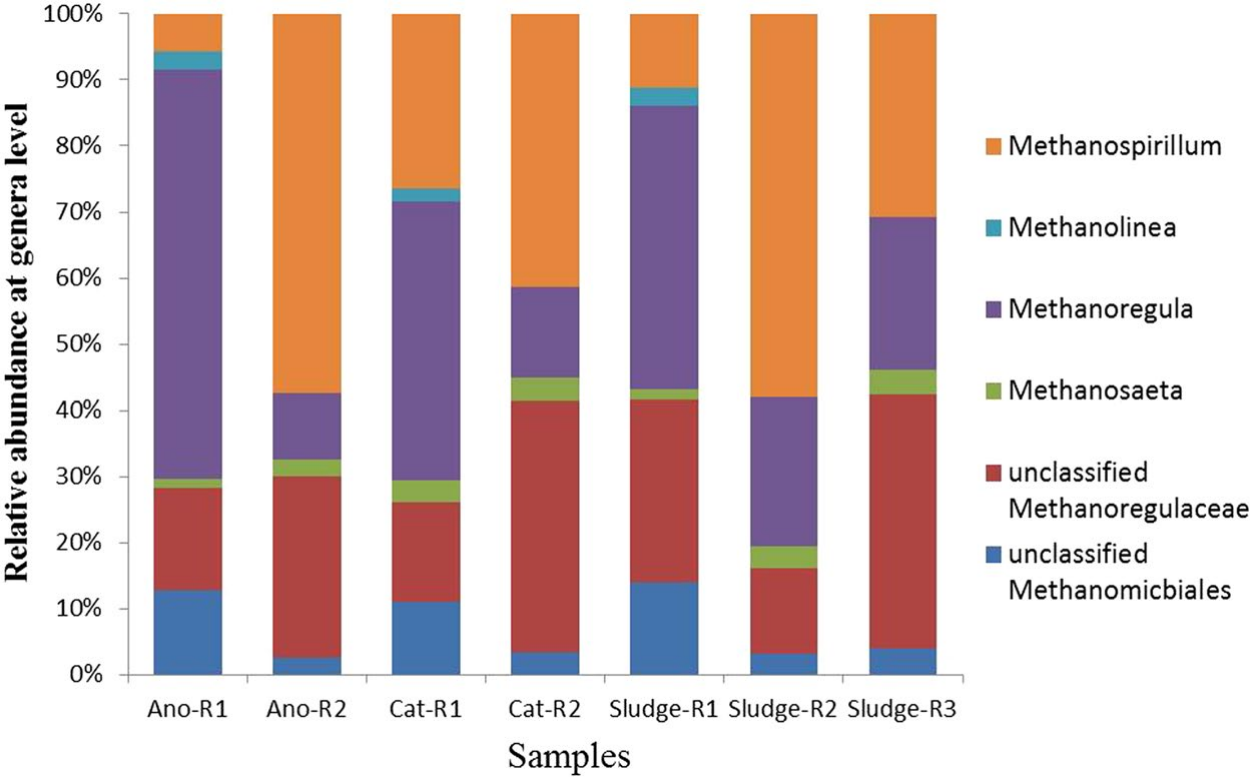
Relative abundance of methanogens in genera level.

For the most anaerobic reactors, the main Archaea are methanogens^24, 33^. As shown in Fig. 3, the dominant methanogen species were different among R1, R2 and R3. In R1, the main methanogen was *Methanoregula*, which constituted approximately 61.11%, 41.98% and 42.47% in the anode, cathode and granular sludge, respectively; in R2, the main methanogen was *Methanospirillum*, which constituted approximately 56.10%, 40.00% and 58.06% in the anode, cathode and sludge, respectively; whereas in R3, the main methanogen was an *unclassified Methanoregulaceae*, which constituted approximately 38.46% in the sludge.

**Figure 4:**
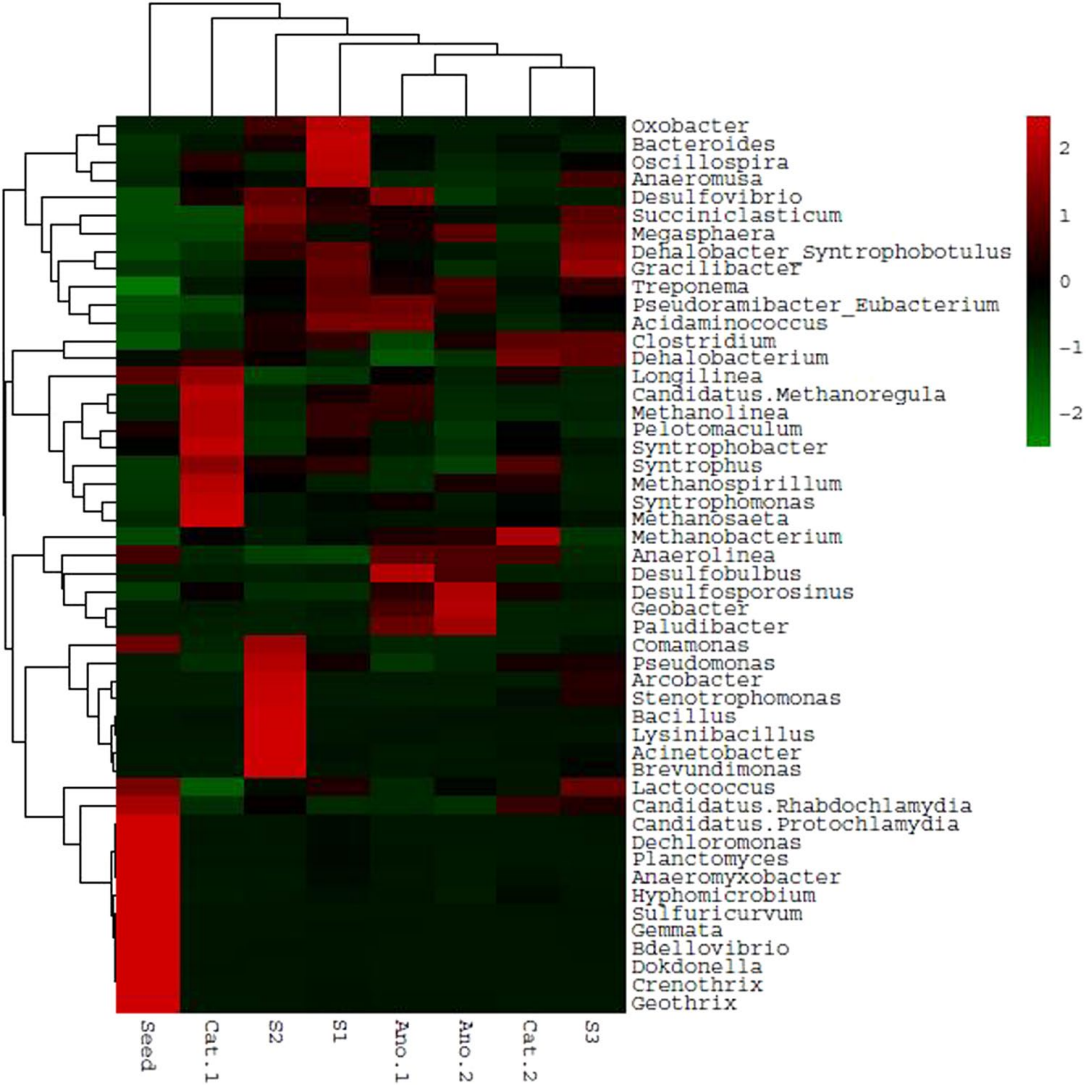
Heatmap taxonomic classification of microbial DNA sequences in R1 (anode, cathode and sludge), R2 (anode, cathode and sludge), R3 (Sludge) and seed sludge at the genera level. The scale bar shows the relative abundance of each genera within a sample.

The microbial communities of the samples from the three reactors were similar in the phylum classification level. The dominant phyla were *Firmicutes*, *Proteobacteria* and *Euryarchaeota*. To further identify the similarities of structure and function of microbial communities among the different parts of the three reactors, a heat map of the microbial community at the genera level was plotted (Fig. 4). The profile revealed that the predominant bacteria in the seed sludge were *Dechloromonas*, *Planctomyces*, *Anaeromyxobacter*, *Hyphomicrobium*, *Sulfuricurvum*, *Gemmata*, *Crenothrix* and *Geothrix*. After cultivation by different systems, the predominant microbial communities exhibited significant change. The samples from the anodes of R1 and R2 showed that the predominant bacteria were potential electroactive species, e.g., *Desulfobulbus*, *Desulfosporosinus*, and *Geobacter*, consistent with previous studies^5, 34^. However, the samples from the cathodes of R1 and R2 differed greatly in microbial communities despite the enrichment of both methanogens. In addition to methanogens, many microorganisms were predominant on Cat-R1, such as *Longilinea*, *Pelotomaculum*, *Syntrophobacter*, *Syntrophus* and *Syntrophomonas*, and they played the important role in degradation of persistent organic pollutants. Conductive magnetite might accelerate these electroactive bacteria for pollutant degradation^16, 30, 35^. Besides, the microbial communities of the samples from the sludge of R1, R2 and R3 were different obviously. The main bacteria in Sludge-R3 were *Succiniclasticum*, *Megasphaera*, *Gracilibacter* and *Dehalobacter Syntrophobotulus*, which could degrade organic matters such as glucose, fatty acids and even organic haloid compounds. The main bacteria in Sludge-R2 were fermentation-related species such as *Comamonas*, *Pseudomonas*, *Arcobacter*, *Stenotrophomonas*, *Bacillus*, *Lysinibacillus* and *Acinetobacter*. In Sludge-R1, the main bacteria were *Oxobacter*, *Bacteroides*, *Oscillospira* and *Anaeromusa*; however, their functions were similar to those in Sludge-R2. The visualized heat map differed in the color pattern among the eight samples, especially for Cat-R1 and Cat-R2, indicating that the addition of magnetite influenced the activity of functional microbes greatly^20, 21^. These results supply a reasonable explanation for the different performance among the three reactors.

#### Enhanced mechanism of combined bioelectrode-UASB with magnetite addition

**Figure 5:**
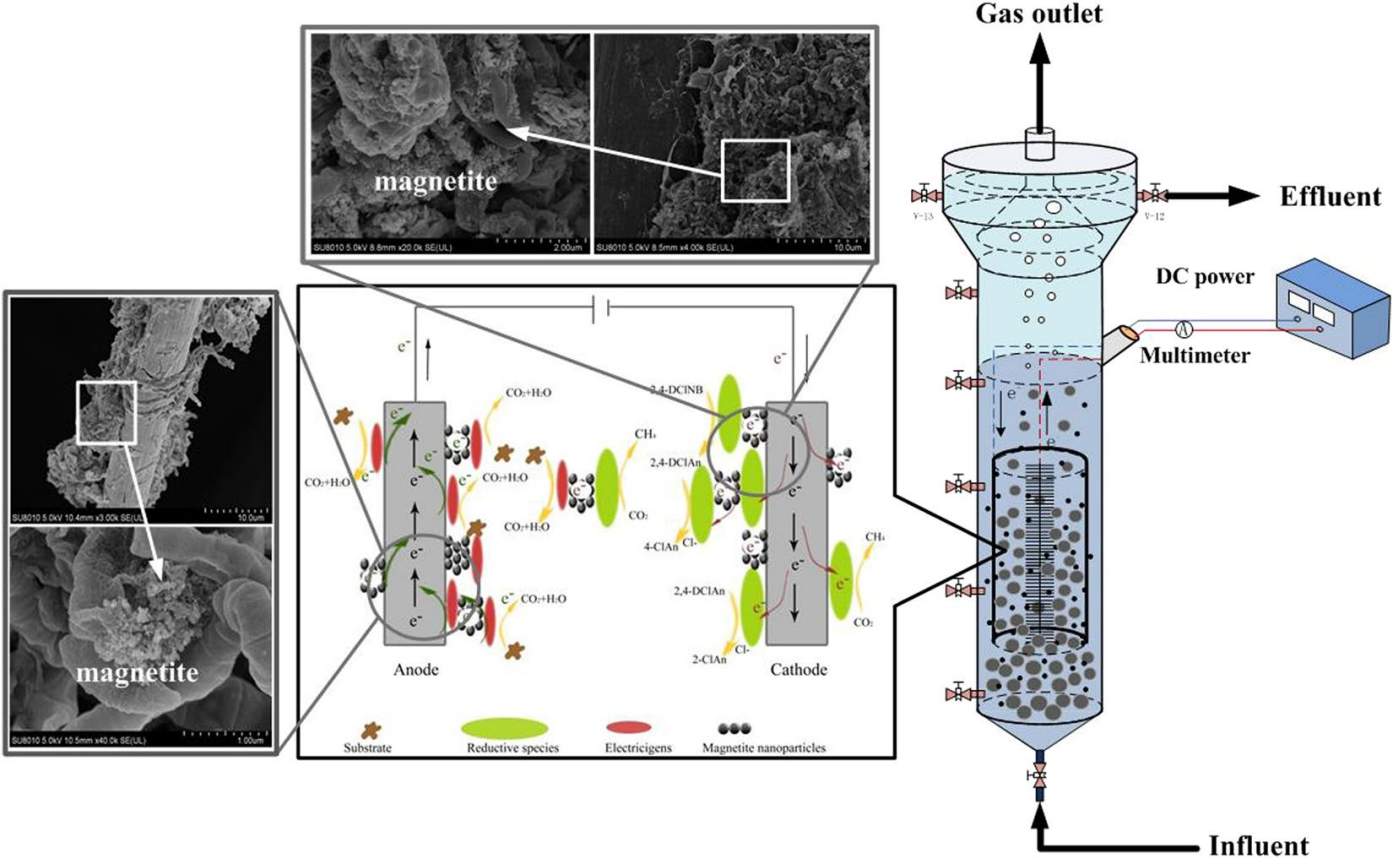
Conceptual illustration of proposed interspecies electron transfer pathway between functional microbes and electrodes in presence of magnetite nanoparticles.

Estevez-Canales *et al*.^36^ showed that iron is essential for the outer-surface c-type cytochrome synthesis, and cytochrome-depleted cells can not reduce Fe(III) citrate or exchange electrons with a graphite electrode, indicating that iron is a key element in electron transfer. Considering the above results analysis and the previous studies^14, 21, 24^, a diagram of the possible enhanced pathways for electron transfer between the microbes and electrodes is proposed in Fig. 5. During the pollutants reduction process, the electron transfer between microbes and cathode could include two pathways. The first one is the 2,4-DClNB-reducing microbes in the cathode biofilm accept the electrons from the cathode surface directly, and then degrade the 2,4-DClNB. The second one is that the 2,4-DClNB-reducing microbes around the cathode use the magnetite as an electron conductor and receive electrons from the cathode surface over a long distance to achieve the reductive transformation of the 2,4-DClNB.

## Conclusions

The combination of magnetite nanoparticles in the bioelectrode-UASB reactor was first investigated in this study. The addition of magnetite nanoparticles not only facilitated the reductive transformation of 2,4-DClNB, but also strengthened the performance of bioelectrode-UASB reactor to resist the shock loading of 2,4-DClNB. Microbial community analysis indicated that more electrogenesis species were enriched with addition of magnetite nanoparticles, and it is speculated that the establishment of cooperative metabolism based on the DIET between dechlorinating microorganisms and electricigens or the electrodes favors the degradation of 2,4-DClNB. In addition, several evidences are emerging with regard to the unique characteristics of electrically conductive iron oxides that accelerate syntrophic cooperation by enhancing direct interspecies electron transfer.

## Materials and Methods

### Electrodes and chemicals

The non-waterproof graphite felt (cathode) and the 160K graphite fiber brush (anode) were purchased from Beijing Sanye Carbon Co. (China).

The 2,4-DClNB (purity > 99.9 %), 2,4-DClAn (purity > 99.9 %), 4-ClAn (purity > 99.9 %), and 2-ClAn (purity > 99.9%) were all analytical grade and purchased from Sinopharm Chemical Reagent Co. (China). Sucrose, KH_2_PO_4_, Na_2_HPO_4_∙3H_2_O, NaHCO_3_ were all analytical grade and purchased from Sinopharm Chemical Reagent Co., Ltd. (China). Methyl alcohol (HPLC grade) was purchased from Sigma-Aldrich Co. (America).

### Synthesis of magnetite particles

The synthesis of magnetite nanoparticles was performed according to a modified protocol^37^. Briefly, Fe_2_(SO_4_)_3_ (6.4 g) and FeSO_4_∙7H_2_O (4.45 g) were dissolved into 0.3 M H_2_SO_4_ solution and then the iron solution was added dropwise into 1.5 M NaOH solution with vigorous stir and black precipitate of Fe_3_O_4_ (magnetite) was synthesized. The precipitate was isolated in a magnetic field, purified by centrifugation (4000 rpm), and suspended in 0.25 L deoxygenated water.

The morphology and particle size of the magnetite nanoparticles were observed by transmission electron microscopy (TEM), and the average size was less than 50 nm (Fig. S9). The dried precipitates were characterized by X-ray diffraction (XRD, Polycrystal X-ray diffractometer of Rigaku Dmax-2550PC, Japan). The XRD pattern obtained was compared with that of pure magnetite.

### Operation of reactors

Three upflow anaerobic sludge blanket (UASB) reactors, namely R1, R2 and R3, were built and operated in parallel. The reactors were constructed with plexiglass with a height of 50 cm and a total volume of 6.6 L. R1 and R2 were installed with a pair of ring bioelectrode; the graphite fiber brush anode (φ2 cm × 14 cm) in the center was surrounded by the graphite felt cathode (φ7 cm × 14 cm), and the spacing between the two electrodes was 2.5 cm. The electrodes in R1 and R2 were installed near the bottom of the reactor and in contact with the sludge blanket; 1 L magnetite colloid solution was added to R1 (total Fe of 3.37 g L^−1^). The electrodes were supplied with a regulated DC power source (Victory3003D, China), and a voltage of 1.5 V was applied according to the previous studies^7, 12^. The schematic diagram of the experimental setup is shown in Fig. S10. R3 was operated as the control. All the reactors were placed in the constant temperature room with temperature controlled at (35 ± 2) °C.

The seeding sludge was collected from a municipal wastewater treatment plant in Hangzhou, China. Each reactor was inoculated with 2.5 L seeding sludge. The mixed liquor suspended solids (MLSS) and the mixed liquor volatile suspended solids (MLVSS) of the seeding sludge were 24.0 g L^−1^ and 16.0 g L^−1^ respectively. The three reactors were fed with synthetic wastewater containing a concentrated stock solution of organic wastewater. The Components of stock solution were as follows (g L^−1^)^38^: Sucrose (90), NH_4_HCO_3_ (14.1), KH_2_PO_4_ (0.97), K_2_HPO_4_∙3H_2_O (1.62), NaHCO_3_ (33.3), and 1 mL of trace element solution was added into 1 L wastewater^39^.

The experiments were divided into two stages. Stage one was the start-up period (70 days), and the reactors were operated under a continuous mode with a hydraulic retention time (HRT) of 24 h. The influent chemical oxygen demand (COD) loading was gradually increased from 0.8 kg m^−3^ d^−1^ to 5.0 kg m^−3^ d^−1^. At stage two the inflow COD loading was maintained at 5.0 kg COD m^−3^ d^−1^ to ensure the stable performance of the reactors, and then 2,4-DClNB was increased gradually from 25 mg L^−1^ to 200 mg L^−1^.

### Analytical methods

Oxidation–reduction potential (ORP), pH, MLSS, MLVSS, COD and dissolved iron ions were determined according to standard methods for the examination of water and wastewater^40^. The electric current was recorded by multimeter. The volatile fatty acids (VFAs) concentration in the effluent and the methane content in the biogas were determined using a gas chromatograph (Agilent 6890N, USA) according to the protocol described by Zhu *et al*.^38^.

The target pollutant of 2,4-DClNB and its intermediate products were determined using a HPLC (Waters 1525, Milford, MA, USA) equipped with a column (Agilent ZORBAX Eclipse SB-C18, Palo Alto, CA, USA), a 717 autosampler and a 2487 dual-wave length UV detector^41^. 1.0 mL samples were centrifuged for 10 min at 10,000 g, and then 0.6 mL of the supernatant was diluted by 0.9 mL methanol and filtered through a 0.22 mm filter membrane. The mobile phase consisted of 55/45 methanol and water (buffered with phosphate at a pH of 2.8) with a flow rate of 1.0 mL min^−1^. The injection volume for all samples was 10 µL, and the column temperature was 25 °C.

The intermediate products were identified by GC-MS (7890B/7000C, Agilent, USA) and LC-MS (6460, Agilent, USA). The sample was pretreated as follows: equivoluminal MtBE was added to the supernatant after centrifugation for 10 min at 10,000 g. Then, the mixed solution was centrifuged for 5 min at 6000 g, and the organic phase was recycled by anhydrous sodium sulfate dehydration. The extraction process was repeated three times. The column temperature of GC-MS was programmed as follows: the temperature was held at 50 °C for 2 min, then increased to 150 °C at 10 °C min^−1^ (1 min), and finally increased to 270 °C at 20 °C min^−1^ (5 min). Afterward, the injection temperature maintained at 250 °C while the interface temperature was maintained at 280 °C. LC-MS was taken on the Agilent 6460 Triple Quad LC/MS using Esi as the ion source, positive/negative ion scanning, a scanning range of (50~800) nm, and a voltage of 100 V.

Cl^−^ was detected by an ion chromatograph (ICS-1100, Dionex, USA) equipped with a guard column (4 × 50 mm, IonPac® AG11-HC), a separation column (4 × 250 mm, IonPac® AS11-HC) and an ECD detector. EGC III KOH was the buffer solution, the injection volume was 25 µL, the flow rate of the mobile phase was 1.0mL min^−1^, and the column temperature was 35 °C.

### Microbial community analysis

Seven biomass samples (Ano-R1, Ano-R2, Cat-R1, Cat-R2, Sludge-R1, Sludge-R2 and Sludge-R3) were collected from the three bioreactors during the steady period. The community structures and distribution of the samples from three bioreactors were characterized by 454 high-throughput sequencing. The DNA of the samples from reactors were extracted using a DNA isolation kit (12888, MoBio PowerSoil). In this experiment, the V4 region (which has a bacterial length of approximately 280 bp) was used for sequencing. Polymerase chain reaction (PCR) amplicon libraries were constructed by the Illumina MiSeq platform using the primers U520F (5′-AYTGGGY DTAAAGNG-3′) and U802R (5′-TACNVGGG TATCTAATCC-3′). The PCR reactions were performed as follows: first 98 °C for 30 s, 25–27 cycles at 98 °C for 15 s, 50 °C for 30 s, 72 °C for 30 s and finally 72 °C for 5 min. A Quant-iT PicoGreen dsDNA Assay Kit was used to quantify the PCR products on a Microplate reader (BioTek, FLx800) and mix the sample according to the amount of required data. The libraries were normalized and quality-controlled using an Agilent High Sensitivity DNA Kit and quantified using the Quant-iT PicoGreen dsDNA Assay Kit. The final sequencing was conducted using a MiSeq Reagent Kit V4 (600 cycles).

### Data availability

The data that support the findings of this study are available from the corresponding author upon request.

## Electronic supplementary material

Supplementary Information
