# Supplementary material for: Magnetite nanoparticles enhance the performance of a combined bioelectrode-UASB reactor for reductive transformation of 2,4-dichloronitrobenzene
Source: Sci Rep. 2017 Sep 4;7:10319. doi: 10.1038/s41598-017-10572-y (PMC5583185; doi:10.1038/s41598-017-10572-y)
Supplement: Supplementary file 1 — Supplementary Information [file 41598_2017_10572_MOESM1_ESM.doc]

**Supplementary Information**

Magnetite nanoparticles enhance the performance of a combined bioelectrode-UASB reactor for reductive transformation of 2,4-dichloronitrobenzene

Caiqin Wang^1^, Lu Ye^1^, Jie Jin^1^, Hui Chen^1^, Xiangyang Xu^1,2^, Liang Zhu^1,2^,*

^1^ *Department of Environmental Engineering, Zhejiang University, Hangzhou 310058, China*

^2^ *Zhejiang Province Key Laboratory for Water Pollution Control and Environmental Safety, Hangzhou 310058, China*

*Corresponding author: Liang Zhu

Address: Department of Environmental Engineering, Zhejiang University, No. 866 Yuhangtang Road, Hangzhou 310058, PR China.

Tel.: +86 571 88982343; Fax: +86 571 88982343

E-mail address: felix79cn@hotmail.com

**Fig. S1 (a) COD removal efficiency and (b) pH change in different reactors during start-up stage**

**Fig. S2 (a) Iron content in the reactors during start-up stage and (b) in the sludge after 70 days running**

**Fig. S3 VFA concentration in the effluent of different reactors after 70 days running**

**Fig. S4 Images of granular sludge on Day 70. a-d: micrograph of granules from seed sludge, R1, R2 and R3 ; e-h: SEM observation of the surface granules from R1; i-j: SEM observation of the inner granules from R1**

**Fig. S5 SEM-EDX images of sludge in R1**

**Fig. S6 (a) Effect of DClNB loading on DClNB removal and (b) balance analysis of effluent pollutants in reactors**

**Fig. S7 SEM photographs of anode in R1 and R2 reactors (a) blank control; (b-d) R1 anode; (e-f) R2 anode**

**Fig. S8 SEM photographs of cathodes in R1(a-b) and R2(c-d) reactors**

**Fig. S9 Transmission electron microscopy of Fe_3_O_4_ particles**

**Fig. S10 Schematic diagram of coupled bioelectrode-UASB reactor**

**Table S1 Cl- formation rates in different reactors**

**Table S2 The observed number of OTUs at a 3% distance**

**Table S3 Alpha diversity index of samples**


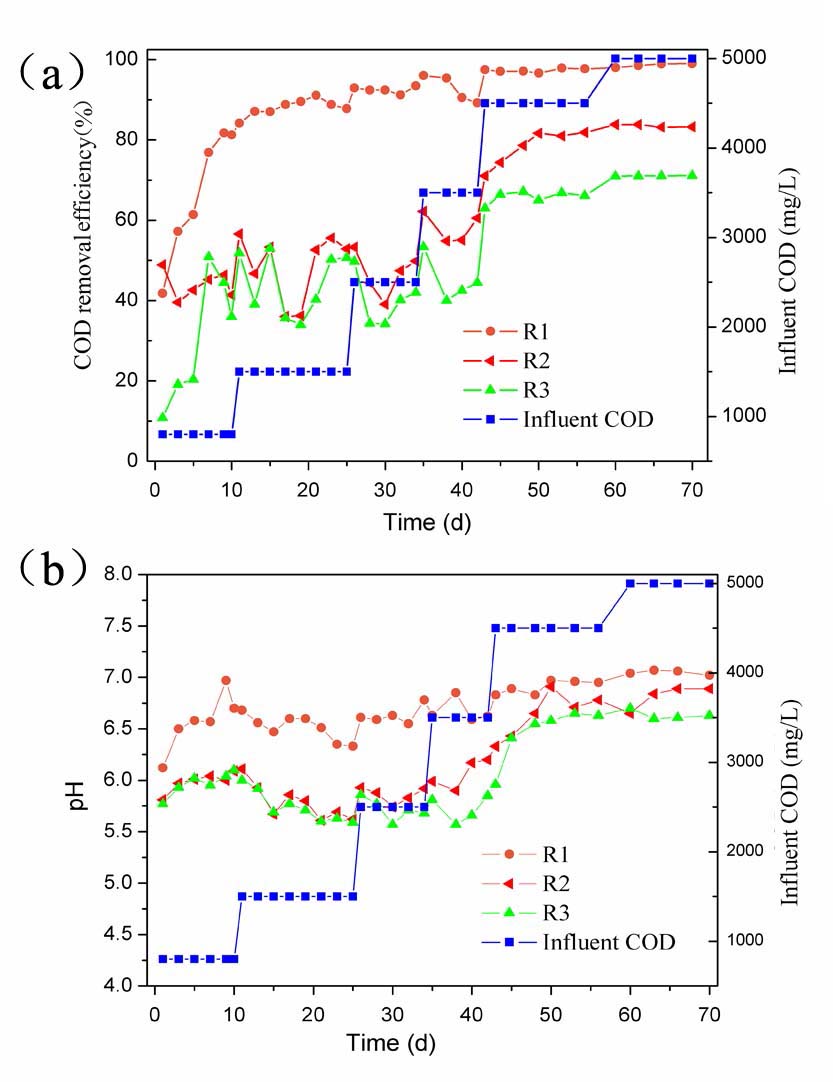


**Fig. S1 (a) COD removal efficiency and (b) pH change in different reactors during start-up stage**


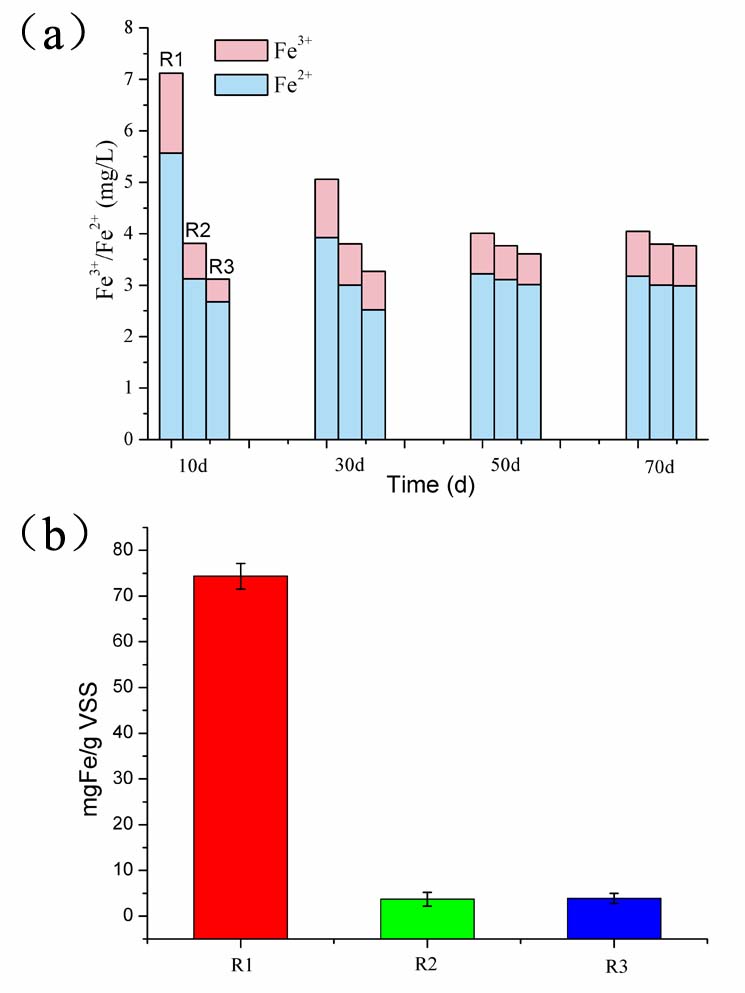


**Fig. S2 (a) Iron content in the reactors during start-up stage and**

**(b) in the sludge after 70 days running**

**Fig. S3 VFA concentration in the effluent of different reactors**

**after 70 days running**


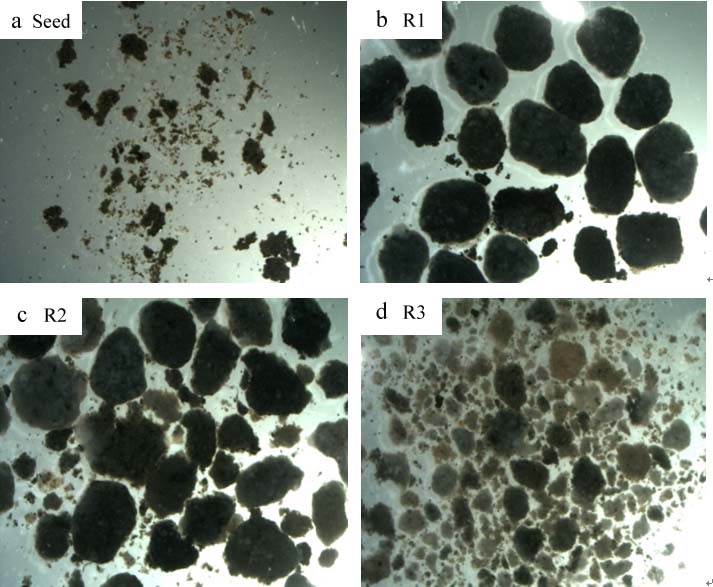


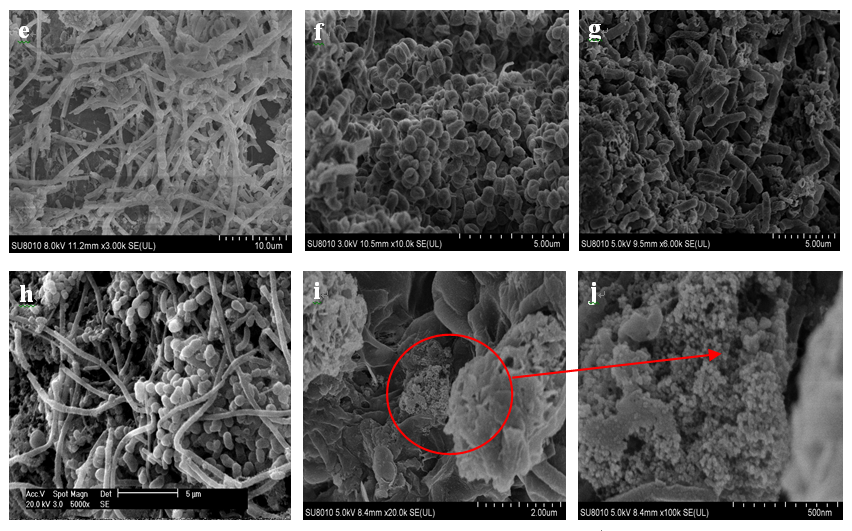
**Fig. S4 Images of granular sludge on Day 70. a-d: micrograph of granules from seed sludge, R1, R2 and R3 ; e-h: SEM observation of the surface granules from R1; i-j: SEM observation of the inner granules from R1**


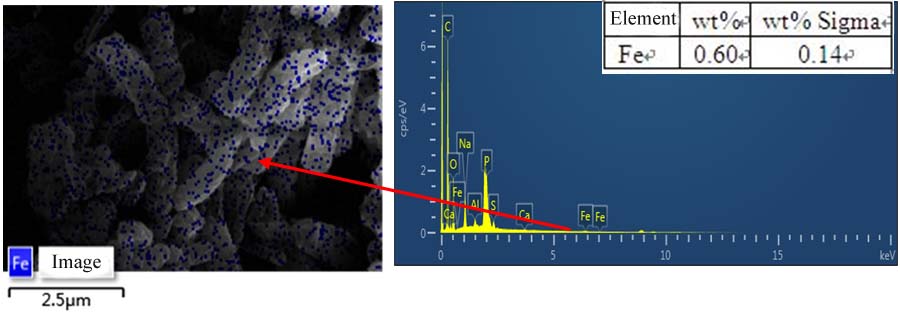


**Fig. S5 SEM-EDX images of sludge in R1**


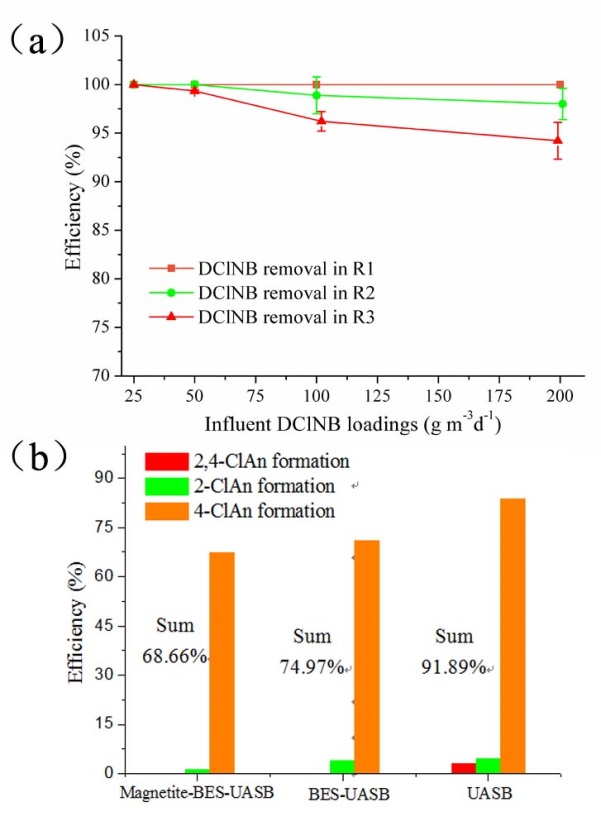


**Fig. S6 (a) Effect of DClNB loading on DClNB removal and (b) balance analysis of effluent pollutants in reactors**


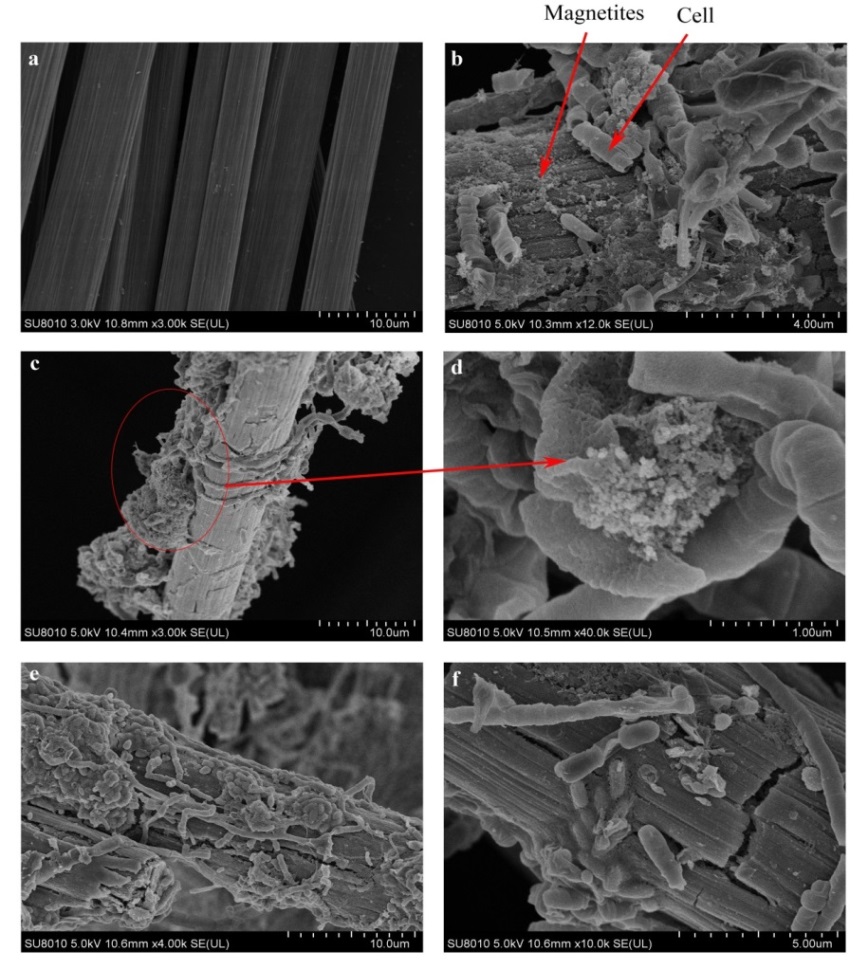


**Fig. S7 SEM photographs of anode in R1 and R2 reactors**

**(a) blank control; (b-d) R1 anode; (e-f) R2 anode**


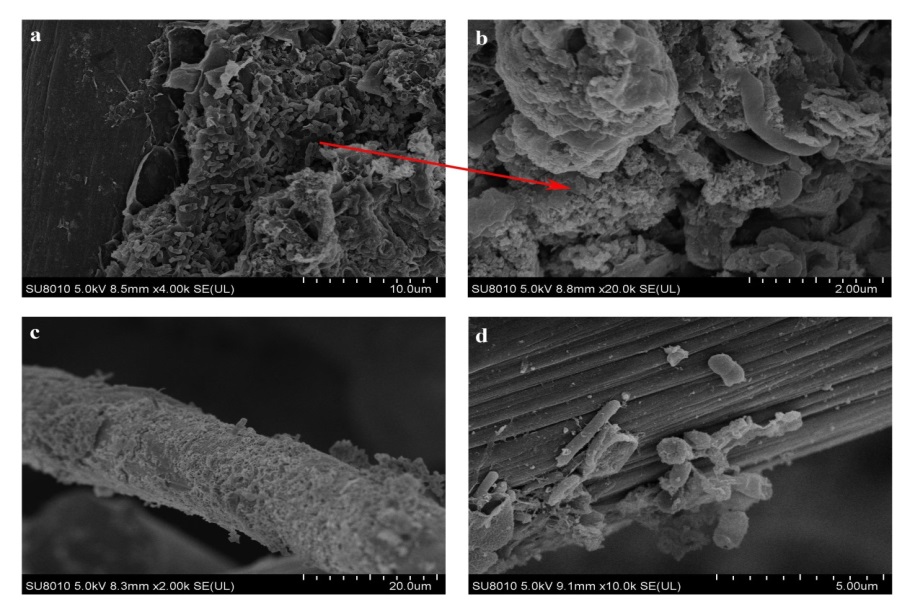


**Fig. S8 SEM photographs of cathodes in R1(a-b) and R2(c-d) reactors**


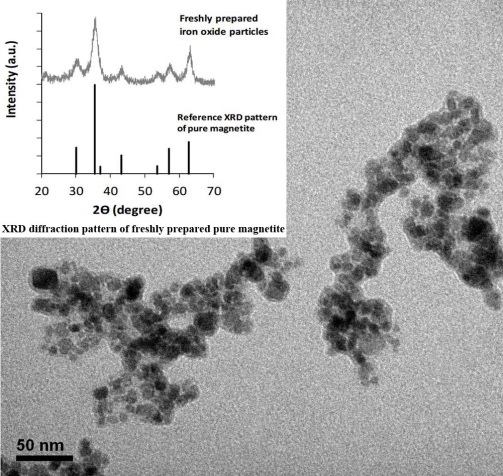


**Fig. S9 Transmission electron microscopy of Fe_3_O_4_ particles**


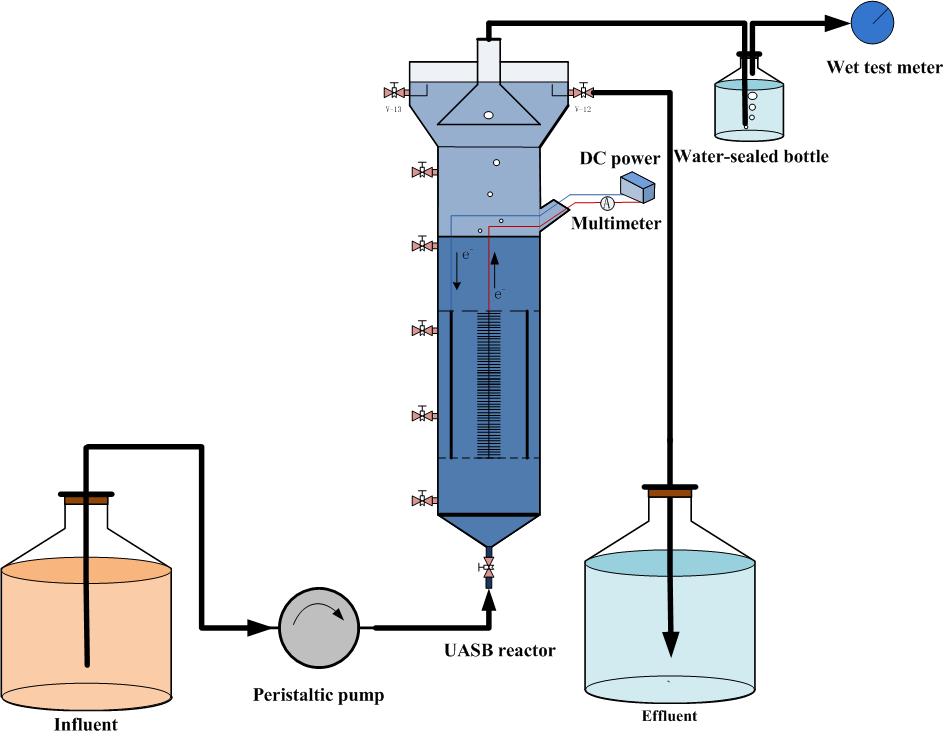


**Fig. S10 Schematic diagram of coupled bioelectrode-UASB reactor**

**Table S1 Cl^-^ formation rates in different reactors**

| Reactors | R1 | R2 | R3 |
| --- | --- | --- | --- |
| Cl^-^ formation(%) | 46.94±1.04 | 42.34±0.96 | 5.43±1.63 |

**Table S**2 The observed number of OTUs at a 3% distance

| Samlpe | Ano-R1 | Ano-R2 | Cat-R1 | Cat-R2 | Sludge-R1 | Sludge-R2 | Sludge-R3 |
| --- | --- | --- | --- | --- | --- | --- | --- |
| OTU | 1580 | 1131 | 1509 | 1187 | 2250 | 1523 | 1016 |

**Table S3 Alpha diversity index of samples**

| Samples | Chao1 | ACE | Simpson | Shannon |
| --- | --- | --- | --- | --- |
| Ano-R1 | 1997 | 1964 | 0.98 | 7.27 |
| Ano-R2 | 1442 | 1419 | 0.92 | 6.14 |
| Cat-R1 | 1864 | 1878 | 0.97 | 7.12 |
| Cat-R2 | 1468 | 1507 | 0.86 | 5.69 |
| Sludge-R1 | 2565 | 2528 | 0.94 | 6.59 |
| Sludge-R2 | 1764 | 1792 | 0.94 | 6.45 |
| Sludge-R3 | 1453 | 1457 | 0.89 | 5.63 |
